# Enhancing recovery and reducing inflammation: the impact of enhanced recovery after surgery recommendations on inflammatory markers in laparoscopic surgery—a scoping review

**DOI:** 10.3389/fsurg.2024.1450434

**Published:** 2024-12-09

**Authors:** Carlos Darcy Alves Bersot, Lucas Ferreira Gomes Pereira, Victor Gabriel Vieira Goncho, José Eduardo Guimarães Pereira, Luiz Fernando dos Reis Falcão

**Affiliations:** ^1^ Department of Anesthesia, BP Hospital – A Beneficência Portuguesa de São Paulo (Anextesia), São Paulo, Brazil; ^2^ Postgraduate in Translational Medicine of the Paulista School of Medicine, EPM-UNIFESP, São Paulo, Brazil; ^3^ Discipline of Anesthesiology, Hospital das Clínicas da Faculdade de Medicina da Universidade de São Paulo, São Paulo, Brazil; ^4^ Department of Anesthesiology, Hospital Unimed Volta Redonda, Rio de Janeiro, Brazil; ^5^ Department of Anesthesiology, Pain and Critical Care Medicine, Federal University of São Paulo (EPM-UNIFESP), São Paulo, Brazil

**Keywords:** enhanced recovery after surgery, inflammation, laparoscopy, anesthesia, surgery

## Abstract

**Introduction:**

The relationship between the Enhanced Recovery After Surgery (ERAS) guidelines and inflammatory markers in laparoscopic surgery has garnered increasing attention. These recommendations are designed to minimize surgical stress and potentially improve recovery outcomes by modifying perioperative care.

**Objective:**

This scoping review aims to evaluate the impact of ERAS recommendations on inflammatory markers in patients undergoing laparoscopic surgeries, identifying current research gaps and consolidating findings from existing studies.

**Methods:**

Guided by the Cochrane Handbook for Systematic Reviews and adhering to the PRISMA-ScR guidelines, this review analyzed studies from databases like PubMed, Scopus, and Cochrane Library. We included both randomized controlled trials and observational studies that assessed inflammatory markers such as C-reactive protein (CRP), white blood cells (WBC), and Interleukin-6 (IL-6) in laparoscopic surgery patients managed with ERAS recommendations.

**Results:**

Out of 64 initial studies, 7 met the inclusion criteria, involving a total of 2,047 patients. Most of the studies focused on laparoscopic colorectal surgeries. Commonly assessed markers were CRP and WBC. The findings consistently showed that ERAS guideline could mitigate the inflammatory response, evidenced by reduced levels of CRP and IL-6, which correlated with fewer postoperative complications and expedited recovery.

**Conclusion:**

ERAS recommendations appear to beneficially modulate inflammatory responses in laparoscopic surgery, which suggests a potential for enhanced recovery outcomes. However, the evidence is currently limited by the small number of studies and inherent methodological biases. Further robust RCTs are required to strengthen the evidence base and refine these protocols for broader clinical application.

**Systematic Review Registration:**

https://osf.io/tj8mw/

## Introduction

The association between inflammation and cancer has been extensively discussed since 1,863, when Virchow observed that tumors frequently develop at sites of chronic inflammation (1). Research has consistently demonstrated that inflammation contributes to tumor growth and aggressiveness; both preoperative and early postoperative inflammatory responses can foster a micrometastatic environment and adversely affect cancer prognosis (1–4).

The initiative to reduce recovery times after surgery was pioneered in the USA under the concept of “fast-track” surgery, particularly aimed at expediting recovery following cardiac procedures (5). Kehlet et al. further advanced this concept by developing a multimodal rehabilitation program focused on colorectal surgeries, which was successful in reducing hospital stay durations (6). The Enhanced Recovery After Surgery (ERAS) programs have refined these preliminary concepts into a standardized, evidence-based approach that enhances surgical outcomes across various disciplines. Originating in Europe, ERAS Society unites diverse surgical teams dedicated to fostering comprehensive, multi-professional patient care (7).

The cellular response to surgical tissue damage triggers the activation of macrophages and neutrophils within the innate immune system via the production of inflammatory cytokines, such as tumor necrosis factor (TNF) alpha, interleukin (IL)-1, and IL-6. These pro-inflammatory cytokines modify the levels of circulating acute-phase proteins, including C-reactive protein (CRP), albumin, ferritin, transferrin, and fibrinogen (8). However, the pathophysiology of post-surgical recovery is not solely a consequence of tissue injury but is inherently multifactorial, encompassing elements such as anxiety, pain, coagulation disorders, hemodynamic changes, and hypoxia. Given the multitude of factors that promote inflammation during the surgical stress response, interventions proposed by the ERAS guidelines address these various components comprehensively (9).

This scoping review aims to provide a descriptive summary of the studies included and to identify potential gaps in the literature regarding the impact of the ERAS guidelines on the inflammatory response following laparoscopic surgery. The guiding question for this review is: “What research has been conducted on the impact of the ERAS recommendations on inflammatory markers in laparoscopic surgery, and what evidence is available regarding its effects on the immune system?”

## Methods

### Study design and protocol registration

This scoping review was guided by the Cochrane Handbook for Intervention Reviews (10) and conducted in accordance with the Preferred Reporting Items for Systematic Reviews and Meta-Analyses extensions for Scoping Reviews (PRISMA-ScR) (11). The research protocol was registered on the Open Science Framework (https://osf.io/tj8mw/).

### Eligibility criteria

We included randomized controlled trials and observational studies assessing the impact of the Enhanced Recovery After Surgery (ERAS) recommendations on inflammatory markers in patients undergoing laparoscopic surgery. Eligible participants were adults aged 18 years or older who underwent any type of laparoscopic surgery and had one or more of the following inflammatory biomarkers measured: C-reactive protein (CRP), white blood cell count (WBC), immunoglobulins (IgG and IgA), tumor necrosis factor (TNF), total protein (TP), cortisol, among others.

The intervention study groups will be considered those adopting any series of measures aimed at optimizing and accelerating recovery in the perioperative period, while the control group will be considered the population with traditional perioperative care. Both nomenclatures will be considered for the intervention group, both ERAS and Fast-track will be accepted.

### Data sources and search strategy

The literature search was conducted using PubMed (MEDLINE), Scopus, Embase, Web of Science, and the Cochrane Controlled Register of Trials (CENTRAL). We also searched for unpublished studies and gray literature through manual searches of reference lists of included articles. The initial search was performed in January 2024, with a follow-up search in February 2024. Searches employed combinations of MeSH terms and their synonyms including “Enhanced Recovery After Surgery,” “Inflammation,” and “Laparoscopy.” Our search strategies, adapted for each database, are detailed in Appendices I and II. No restrictions were placed on language or publication date.

### Study selection

Three reviewers (VGVG, LFGP, CDAB) independently screened titles and abstracts using a standardized screening protocol. Full texts of potentially relevant studies were retrieved, and their details uploaded into Rayyan® (Qatar Computing Research Institute, Doha, Qatar) (12). Disagreements among reviewers were resolved through discussion or consultation with a third party (JEGP, LFRF).

### Data extraction and risk of bias assessment

Data were extracted independently by the same four reviewers using a specially developed form. Extracted information included publication year, country, study type, surgical type, population characteristics, ERAS recommendations details, inflammatory markers, and pertinent findings. Authors were contacted to resolve data discrepancies or clarify missing details.

Risk of bias was assessed using the Guyatt-modified Cochrane approach for randomized trials (13, 14) and the Morgan approach for non-randomized studies (15). The criteria for randomized trials included adequacy of random sequence generation, allocation concealment, blinding (of investigators, patients, data collectors, statisticians, outcome assessors), completeness of outcome data, and absence of selective reporting. A threshold of less than 10% total loss to follow-up was considered low risk. Non-randomized studies were assessed for eligibility criteria, outcome and exposure measurement accuracy, confounder control, and follow-up adequacy.

### Data analysis and presentation

Data were synthesized and displayed in two tables highlighting the characteristics of the ERAS recommendations and the inflammatory markers assessed. Details such as publication year, country, study type, surgical type, ERAS details, and outcomes were tabulated. If the outcomes found and summarized in the results are amenable to quantitative analysis, they will be analyzed by means of a meta-analysis, divided into different outcomes.

## Results

### Search results and study selection

**Figure 1:**
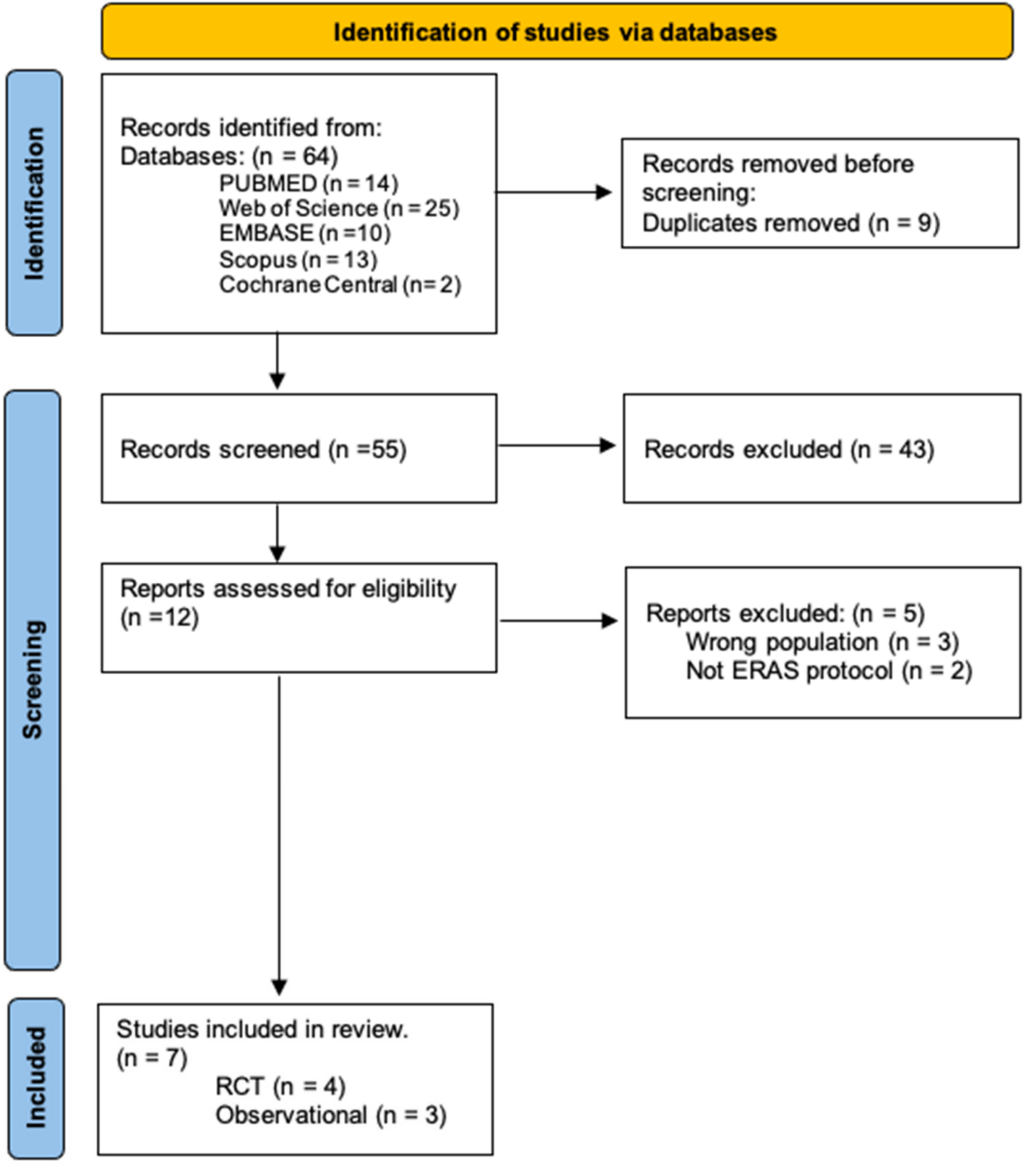
PRISMA flowchart.

Our systematic database search initially identified 64 studies. After removing 9 duplicates, 55 records were screened by title and abstract, with 43 subsequently excluded. Full texts of the remaining 12 studies were evaluated for eligibility, resulting in 5 further exclusions. Ultimately, 7 studies met our inclusion criteria. The selection process is depicted in the PRISMA flowchart (Figure 1).

### Characteristics of included studies

The included studies were published between 2012 and 2022. Four of these were randomized clinical trials, and three were non-randomized observational studies (Table 1). The studies involved both male and female participants, with the ERAS group having a mean age of 59.7 years-old compared to 49.7 years-old in the control group. Geographically, four studies were conducted in China (16–19), one in Italy (20), and one in South Korea (21) (Tables 2, 3). The surgical interventions examined included colorectal surgeries (16–18, 20, 21), gastrectomy (19), and gynecological oncological surgery (22).

**Table 1:** Study characteristics according to population and type of publication.

Authors	Year and Country	Type of study	Type of surgery	Population
Xu et al.	2015, China	Randomized Controlled Trial	Colorectal laparoscopic	92 patients
Wang et al.	2012, China	Randomized Controlled Trial	Colorectal laparoscopic	163 patients
Tian et al.	2020, China	Retrospective cohort	Laparoscopic Gastrectomy	1,026 patients
Peng et al.	2021, China	Randomized Controlled Trial	Gynecological Oncology	130 patients
Mari et al.	2016, Italy	Randomized Controlled Trial	Colorectal laparoscopic	140 patients
Liu et al.	2020, China	Retrospective cohort	Colorectal laparoscopic	200 patients
Jalloun et al.	2020, South Korea	Retrospective cohort	Colorectal laparoscopic	296 patients

**Table 2:** Study characteristics related to population and setting.

Author	Country/Year	Participants included	Mean age per study group	Male gender per group	Inclusion criteria	Exclusion criteria	Follow-up time
Jalloun et al.	2020, South Korea	296 patients I-82 C-214	I-<65 37 (45.1) ≥65 45 (54.9) C-<65 100 (46.7) <65 114 (53.3)	I-44 C-127	All patients with diagnostic colonic cancer	–	4 days
Liu et al.	2020, China	200 patients I-100 C-100	I-68.49 C-65.95	I-57 C-48	(1) pathologically confirmed CRC; (2) treated with one-stage radical resection (excluding Miles procedure) by the same group of doctors; (3) good nutritional status; (4) no significant heart, lung, kidney, or other important organ dysfunction; (5) no distant tumor metastasis; (6) no previous abdominal surgery; (7) no radiotherapy or chemotherapy; and 8) anesthesia ASA score <4 points.	(1) patients with such intestinal as obstruction or perforation requiring immediate surgery; (2) severe malnutrition; (3) poor mobility; (4) anesthesia ASA score >4 points; and (5) mental illness.	9 h
Mari et al.	2016, Italy	140 patients I-70 C-70	I-64 (42–83) C-67 (39–87)	I-39 C-35	Patients between 18 and 80 years of age, with American Society of Anesthesiologists grades I through III, autonomous for mobilization and walking, eligible for laparoscopic technique	–	5 days
Xu et al.	2015, China	92 patients I-46 C-46	I-59.3 ± 12.5 + 59.1 ± 9.8/2 C-58.0 ± 13.2 + 60.8 ± 7.6/2	I-29 C-28	(1) ASA I–III (no life-threatening systemic diseases) (2) Age ≥18 years (3) With pathologically confirmed colon and upper rectal cancer.	(1) Patients are younger than 18 years. (2) ASA grade ≥IV (3) Preoperative evidence of distant metastases (4) History of malignant disease (5) Tumors can be resected by endoscopic mucosal resection (EMR) or endoscopic submucosal dissection (ESD), bowel obstruction or perforation, and patients undergoing total colectomy, mid-low rectal cancer, and pregnancy.	4 days
Wang et al.	2012, China	163 patients I-81 C-82	I-56 C-58	I-51 C-51	The inclusion criteria were as follows: no disease of the immune system; no preoperative radiotherapy or chemotherapy. No history of operation on abdominal and distant metastases; ASA score: degree I–III; and self-care function prior to hospitalization.	The exclusion criteria were as follows: association with other organ resection, conversion from laparoscopic operation to laparotomy, inability to place an epidural catheter, inability to infuse drugs, need for a stoma, and emergency operation.	Blood samples were obtained on the day before operation, as well as on days 1, 3, and 5 after operation.
Tian et al.	2020, China	1,026 patients	I-59.6 C-59.4	I-279 C-298	Laparoscopic gastrectomies were performed at the Department of General Surgery of the Affiliated Hospital of Qingdao University, China from January 2012 to December 2015.	Not specified the exclusion criteria.	5 years
Peng et al.	2021, China	130 patients I-65 C-65	I-47,4 C-43.53	I–0 C–0	Patients between the age of 18–70 years, who were diagnosed with cervical tumors, uterine tumors or ovarian tumors, were eligible for enrollment.	-had a history of constipation and severe comorbidity, including patients with American Society of Anesthesiologists risk ≥4, severe organ dysfunction or failure, a comorbidity-polypharmacy score ≥22	6 months

**Table 3:** Assessment of the ERAS protocol and the inflammatory markers analyzed.

Authors and Year	Type of surgery	Groups studied	ERAS protocol	Inflammatory markers	Results
Jalloun et al., 2020	Colorectal laparoscopy	ERAS protocol group (EP) and Control protocol group (CP).	*Preoperative:* patient education, oral carbohydrate treatment, formula intake, thrombosis prophylaxis, antibiotics prophylaxis. *Intraoperative:* Epidural or spinal anesthesia, body temperature preservation, restrictive fluid strategy, PONV prophylaxis. *Postoperative:* Epidural analgesia, effective pain control, balanced fluids, stimulation of gut motility, termination of urinary drainage, termination of IV fluid infusion, mobilization, and energy intake.	White blood count (WBC); C-reactive protein level; albumin level; neutrophil/lymphocyte ratio (NLR), and the time required after surgery for the leukocyte count to drop below 10,000/mm^3^ in days.	Increased WBC count: EP = 42,7%; CP = 72,9; Time required for the WBC count to normalize was significantly shorter in the EP group than in the CP group (*P* ≤ 0.001). C-reactive protein level: EP = 24,1%; CP = 80,6% (*P* < 0.001).
Liu et al., 2020		Traditional treatment (TT) and ERAS group (EG).	*Preoperative:* patient education, gastrointestinal preparation. *Intraoperative:* Body temperature, liquid management (esophageal doppler ultrasound). *Postoperative:* Analgesia (continuous epidural), catheter indwelling, early enteral nutrition and early activities.	The blood was collected 24 h before surgery, to obtain the preoperative NLR. Another blood sample was collected on the postoperative day 3 to calculate NLR. Total protein (TP) and albumin (alb) were also analysed.	Preoperative NLR: TT = 2.96 ± 0.98; EG = 3.03 ± 0.92; Postoperative NLR: TT = 3.71 ± 0.68; EG = 3.22 ± 0.85; Preoperative TP: TT = 71.40 ± 5.36; EG = 70.09 ± 6.12; Postoperative TP: TT = 52.92 ± 1.73; EG = 57.82 ± 2.27; Preoperative alb: TT = 45.78 ± 3.67; EG = 44.94 ± 3.80. Postoperative alb: TT = 32.83 ± 1.69; EG = 37.07 ± 1.46.
Mari et al., 2016		ERAS group (EG) and Standard group (SG).	*Preoperative:* Bowel preparation, 200 ml oral maltodextrin intake 6 and 2 h before surgery. *Intraoperative:* Fluid restriction (5–10 ml/kg/h), Nasogastric tube removal. *Postoperative:* Spinal analgesia and opioid and NSAID oral, fluid management (1,500 ml/d), fluid meal after 6 h and solid meal after 24 h and mobilization 6 h after surgery.	The blood was collected preoperatively, 1, 3 and 5 days after surgery. Cortisol, C-reactive protein (CRP), WBC count, interleukin (IL)-6;	Preoperative CRP: EG = 6.2 ± 8.9; SG = 14.1 ± 16.4; Day 1 CRP: EG = 50.9 ± 14.5; SG = 93.2 ± 11.3; Day 3 CRP: EG = 53.2 ± 13.1; SG = 89.8 ± 14.6; Day 5 CRP: EG = 36.3 ± 19.5; SG = 78.6 ± 10.3. Preoperative WBC: EG = 6,486 ± 2,353; SG = 6,472 ± 1,821; Day 1 WBC: EG = 9,717 ± 2,869; SG = 9,598 ± 2,950; Day 3 WBC: EG = 8,325 ± 2,706; SG = 8,947 ± 2,943; Day 5 WBC: EG = 6,670 ± 2,317; SG = 7,321 ± 2,349. Preoperative IL-6: EG = 11.3 ± 6.9; SG = 6.3 ± 9.1; Day 1 IL-6: EG = 20.6 ± 8.2; SG = 39.2 ± 12.1; Day 3 IL-6: EG = 17.8 ± 9.9; SG = 41.8 ± 12.4; Day 5 IL-6: EG = 14.3 ± 8.8; SG = 35.9 ± 10.3. Preoperative cortisol: EG = 16.6 ± 6.3; SG = 16.5 ± 5; Day 1 cortisol: EG = 12.8 ± 7.8; SG = 15.9 ± 9.4; Day 3 cortisol: EG = 19 ± 9.2; SG = 19.6 ± 7.6. Day 5 cortisol: EG = 17.4 ± 6.96; SG = 21 ± 8.7.
Xu et al., 2015		Laparoscopy with fast-track treatment (LAFT); Laparoscopy with conventional treatment (LAC)	*Preoperative:* Oral carbohydrates before surgery. *Intraoperative:* Fluid restriction, body warming. *Postoperative:* early oral nutrition, early ambulation and early removal of nasogastric tube.	The blood samples were collected preoperatively, 12 h and 96 h after surgery. IgG, IgM, IgA, T and NK cells were evaluated.	Preoperative IgG: LAFT =91.6 (10.5); LAC 90.2 (10.1); 12 h IgG: LAFT = 88.2 (72.6–101.2); LAC = 87.6 (72.0–101.3); 96 h IgG: LAFT = 94.9 (80.1–121.7); LAC = 92.7 (70.8–109.0). Preoperative IgA: LAFT = 93.4 (11.6); LAC = 92.1 (11.3); 12 h IgA: LAFT = 88.7 (70.3–120.8); LAC = 88.6 (74.3–106.3); 96 h IgA: LAFT = 98.2 (80.1–115.7); LAC = 95.5 (73.3–111.6). Preoperative IgM: LAFT = 90.3 (17.6); LAC = 90.2 (34.2); 12 h IgM: LAFT = 87.6 (49.0–116.0); LAC = 85.4 (36.9–113.7); 96 h IgM: LAFT = 93.1 (64.6–145.9); LAC = 95.1 (34.5–271.2). Preoperative T cells: LAFT = 94.7 (16.4); LAC = 95.4 (20.9); 12 h T cells: LAFT = 86.8 (57.6–128.3); LAC = 84.6 (53.8–103.4); 96 h T cells: LAFT = 102.6 (79.5–126.0); LAC = 106.2 (57.5–152.9). Preoperative NK cells: LAFT = 122.4 (57.2); LAC = 119.5 (44.4); 12 h NK cells: LAFT = 147.7 (39.8–324.3); LAC = 137.0 (90.2–246.0). 96 h NK cells: LAFT = 97.2 (50.9–158.5); LAC = 102.1 (43.4–188.9).
Wang et al., 2012		Fast-track laparoscopic group (FTL); Traditional protocol laparoscopic group (TL).	Preoperative: no bowel preparation, 10% glucose injection orally administered; intake of oral fluids until 2 h before initiation of surgery and 6 h fast for solid food. Intraoperative: General anesthesia with epidural catheter, no surgical drains. Postoperative: Use of epidural catheter, discard urinary catheterization within 24 h, early deambulation and free fluids on the operation day, followed by regular diet on the other day.	The blood samples were collected on the preoperative, post-operative day 1, day 3 and day 5. The inflammatory markers analysed were: Serum C-reactive protein (CRP) levels, IL-6, CD3; CD4 and CD4/CD8 ratio.	Preoperative CRP: FTL = 4.83 ± 3.76; TL = 4.48 ± 3.05; CRP Day 1: FTL = 60.52 ± 19.1; TL = 87.21 ± 16.05; CRP Day 3: FTL = 84.45 ± 15.31; TL = 99.55 ± 14.46; CRP Day 5: FTL = 54.65 ± 15.03; TL = 72.85 ± 14.95. Preoperative IL-6: FTL = 19.82 ± 8.11; TL = 19.16 ± 8.14; IL-6 Day 1: FTL = 100.29 ± 19.43; TL = 135.35 ± 15.53; IL-6 Day 3: FTL = 70.58 ± 12.13; TL = 95.26 ± 13.55; IL-6 Day 5: FTL = 45.65 ± 8.25; TL = 60.43 ± 10.54. Preoperative CD3: FTL = 55.21 ± 2.77; TL = 55.15 ± 2.65; CD3 Day 1: FTL = 47.81 ± 3.27; TL = 46.13 ± 2.13; CD3 Day 3: FTL = 50.35 ± 3.02; TL = 47.61 ± 2.34; CD3 Day 5: FTL = 51.92 ± 2.65; TL = 49.21 ± 2.29; Preoperative CD4: FTL = 33.02 ± 3.22; TL = 32.95 ± 3.59; CD4 Day 1: FTL = 27.05 ± 2.34; TL = 27.12 ± 3.01; CD4 Day 3: FTL = 28.02 ± 2.13; TL = 28.05 ± 2.19; CD4 Day 5: FTL = 30.38 ± 2.94; TL = 29.02 ± 2.41. Postoperative CD4/CD8 ratio: FTL = 1.24 ± 0.24; TL = 1.22 ± 0.25; CD4/CD8 ratio day 1: FTL = 1.05 ± 0.22; TL = 0.94 ± 0.19; CD4/CD8 ratio day 3: FTL = 1.11 ± 0.24; TL = 1.03 ± 0.21; CD4/CD8 ratio day 5: FTL = 1.14 ± 0.20; TL = 1.06 ± 0.22.
Tian et al., 2020	Laproscopic Gastrectomy	ERAS Group (EG) and Conventional group (CG)	*Postoperative:* Patient education, organ function evaluation and pre-rehabilitation, 6 h fasting and 2 h drinking. *Intraoperative:* No indwelling nasogastric tube, precision surgery scheme, goal-directed therapy, epidural anesthesia, heat preservation, small midline incision, multimodal analgesia. *Postoperative:* Mobilization on the postoperative day, oral diet on the first operative day, early removal of catheter and early extraction of abdominal drainage.	Preoperative, postoperative day 1, postoperative day 4 and postoperative day 6. White Blood Count (WBC); C-reactive protein (CRP), and procalcitonin.	The results were not quantitatively available in absolute numbers, only a graphical representation. On the results the inflammatory indexes are cited on the text: he CRP (0.63 ± 0.33 vs 0.58 ± 0.30) and procalcitonin (90.61 ± 20.42 vs 78.35 ± 16.73) levels on POD 3/4 were significantly different between the two groups (*P* < 0.001).
Peng et al., 2021	Gynecological Oncology	ERAS Group (EG) or Conventional group (CG).	*Before admission:* Preoperative education and operation risk assessment; *Preoperative*: no bowel preparation, fasting up to 6 h before surgery, carbohydrate 2,5% up to 2 h before surgery. *Day of surgery:* Insertion of foley catheter, antiembolic stockings, fluid restriction (4–5l); Multimodal analgesia with injection of bupivacaine in transabdominal surgery after incision closure; encourage ambulation and sip of water 2 h later after surgery. *Postoperative:* Low molecular weight heparin injection, foley and drain removal as early as possible, encourage ambulation, fluid restriction (1–2l); NSAIDs IV for 3 days and semifluid diet in the first postoperative day.	The blood samples were collected at the preoperative period and the time it was collected at the postoperative period was not specified. White blood count (WBC), neutrophils, lymphocytes, monocytes and platelets count.	The results were not quantitatively available in absolute numbers, only a graphical representation. But at the results text they recognize that at the enhanced recovery pathway patients are significantly lower compared with the conventional group

### ERAS recommendations characteristics

The ERAS recommendations varied but commonly included preoperative patient education, a 6 h fasting period, and carbohydrate-rich liquids up to 2 h before surgery. Intraoperative measures focused on fluid restriction, body temperature maintenance, and multimodal anesthetic strategies, including epidural anesthesia in four studies. Postoperative care emphasized early mobilization across all studies.

### Sampling and inflammatory markers

Blood samples were collected at various times: six studies (16–20, 22) during the intraoperative period, one study (17) 12 h post-surgery, 4 studies (18–21) collected on the first postoperative day, 3 studies (16, 18, 20) collected on the 3rd postoperative day, 2 studies (17, 19) collected on the 4th postoperative day and 2 studies (18, 20) on the 5th postoperative day, and Tian et al. (19) was the only study to analyze samples on the 6th postoperative day. Inflammatory markers analyzed included white blood count (WBC) (19–22), C-reactive protein (CRP) (18–21), albumin (16, 21), neutrophil/lymphocyte ratio (NLR) (16, 21, 22), total protein (TP) (16), cortisol (20), interleukin-6 (IL-6) (18, 20), immunoglobulin (Ig) G, IgM, IgA (17), T lymphocytes (17, 18), natural killer (NK) cells (17), procalcitonin (19), and platelet count (22) (Table 3).

### Risk of bias in studies

Randomized studies showed adequate random sequence generation and allocation concealment. Blinding of participants was achieved, but not for surgical staff due to the nature of the procedures. We did not consider this at high risk of bias because those outcomes cannot be influenced by the participants. Blinding of caregivers was considered at high risk due to the impossibility to hide surgical technique from them. Blinding of data collectors, statisticians, and outcome assessors were considered at low risk of bias in all studies. There were no studies reporting total loss to follow-up above the 10% threshold nor above 5% between groups. Therefore, loss to follow-up was considered as low risk of bias (Table 4).

**Table 4:** Risk of bias randomized studies.

Author/Years	Was the randomization sequence adequately generated?	Was allocation adequately concealed?	Was there blinding of participants?	Was there blinding of the caregiver?	Was there blinding of data collectors?	Was there blinding of staticians?	Was there blinding of outcome assessors?	Was loss to follow-up (missing outcome data) infrequent?*	Are reports of the study free of suggestion of selective outcome reporting?	Was the study apparently free of other problems that could put it at a risk of bias?
Xu et al. (2015)	Definetely yes	Probably yes	Probably not	Definetely not	Probably yes	Probably yes	Definetely yes	Definetely not	Probably yes	Definetely yes
Wang et al. (2012)	Definetely yes	Probably yes	Probably not	Definetely not	Probably yes	Probably yes	Definetely yes	Definetely not	Probably yes	Definetely yes
Peng et al. (2021)	Definetely yes	Probably yes	Probably not	Definetely not	Probably yes	Probably yes	Definetely yes	Definetely not	Probably yes	Definetely yes
Mari et al. (2016)	Definetely yes	Probably yes	Probably not	Definetely not	Probably yes	Probably yes	Definetely yes	Definetely not	Probably yes	Definetely yes

Non-randomized studies showed critical. Bias due to confounding was considered critical in two studies (19, 21), and considered to be serious in one study (19), because they did not correct the adequate non-exposed cohort groups for confounding factors. Bias in selection of participants was considered to be serious in all observational studies (16, 19, 21), because selection was offered, and not encompassing all the patients. Bias in classification of exposures was considered moderate in three studies (16, 19, 21) because information was self-reported. Bias due to missing data was considered serious in all studies (16, 19, 21) due to the design of the studies (Tables 5, 6).

**Table 5:** Risk of bias non-randomized studies.

Author year	Do exposed individuals represent the general population?	Was certainty of exposure adequate?	Was selection of non-exposed cohort adequate?	Demonstration that outcome of interest was not present at start of study?	Comparability of cohorts (age)	Comparability of cohorts (other controlled factors)	Assessment of Outcome	Adequate follow-up time	Loss to follow-up
Categories	SELECTION (1 POINT PER QUESTION)			COMPARABILITY (1 POINT PER QUESTION)			OUTCOMES (1 POINT PER QUESTION)		
Tian (2000)	yes	Yes	No	Yes	Yes	Yes	Yes	No	Yes
Liu (2020)	yes	Yes	Yes	Yes	Yes	Yes	Yes	No	Yes
Jalloun (2020)	yes	Yes	Yes	Yes	Yes	Probably yes	Yes	Yes	Yes

**Table 6:** Assessment of GRADE.

Quality assessment					Summary of findings			Certainty in estimates
					Average (CI 95%)	Anticipated absolute effects		
N^o^ of participants (studies) Follow-up in days	Risk of bias	Inconsistency	Indirectness	Imprecision	Publication bias	Control^a^	ERAS	
CRP postoperative day three								
220 (2) 5 days	No serious limitations	Serious limitations^a^	Serious limitations^b^	Serious imprecision^c^	Undetected	Mean CRP reduction was −25.98	Average 25.98 less (−47.05 less to −4.91 less).	⊕*ΟΟΟ* VERY LOW
CRP postoperative day five								
220 (2) 5 days	No serious limitations	Serious limitations^a^	Serious limitations^b^	Serious imprecision^c^	Undetected	Mean CRP reduction was −28.50	Average −28.50 less CRP (−55.46 less to −1.53 less)	⊕*ΟΟΟ* VERY LOW

^a^ There was a serious limitation related to inconsistency (*I*^2^ > 50%).

^b^ There was a serious limitation related to indirectness (not a clinical outcome.

^c^ There was a serious limitation related to imprecision (rated down twice due to low number of events and wide confidence intervals).

### Outcomes

**Figure 2:**
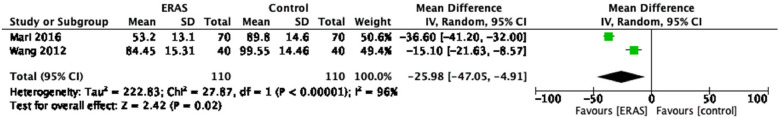
Comparison of CRP on postoperative days three between ERAS and control groups.

C-reactive protein (CRP): has been investigated in two observational studies (19, 21) and two RCTs (18, 20). The studies included 1,322 patients in the retrospective cohorts (19, 21) and 303 patients in two RCTs (18, 20). It found significantly lower levels of CRP in ERAS group when compared with standard on postoperative day (POD) 1, 3, and 5. All retrospective studies found lower plasma concentrations of CRP after laparoscopy when compared to open surgery. Jelloun et al. (21), 2020, including 296 patients, found an increase in CPR count of 24.1% in the ERAS group and an increase of 80.6% in the control group (*p* < 0.001). Tian et al. (19), 2020, including 1,026 patients, found no significant reduction of CRP in the ERAS group compared to the control group. Mari et al. (20), 2016, including 140 patients, found that ERAS protocol significantly reduced the rise slope of CRP on postoperative days, 1, 3, and 5 (*p* < 0,05) compared to the control group. Results from two RCTs, including 220 participants, found a significant reduction on the rising slope of C-reactive protein on postoperative day three in the ERAS group compared to the control group (MD: −25.98, 95% CI: −47.05, −4.91; *p* = 0.02; *I*^2^ = 96%). Certainty of evidence was considered very low due to imprecision (wide confidence interval and low number of patients), inconsistency (from high heterogeneity) and indirectness (Figure 2).

**Figure 3:**
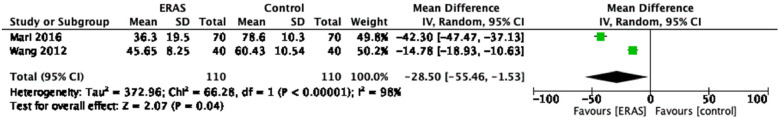
Comparison of CRP on postoperative days five between ERAS and control groups.

Results from two RCTs (18, 20), including 220 participants, found a significant reduction on the rising slope of C-reactive protein on postoperative day five in the ERAS group compared to the control group (MD: −28.50 95% CI: −55.46, −1.53; *p* = 0.04; *I*^2^ = 98%). Certainty of evidence was considered very low due to imprecision (wide confidence interval and low number of patients), inconsistency (from high heterogeneity) and indirectness (Figure 3).

*White blood cell count (WBC)*: two observational (19, 21) and two RCT (20, 22) analyzed this inflammatory marker. They found significant reduction of WBC in the ERAS group compared to control on POD 1, 3, and 5. Jaloun et al. (21), 2020, including 296 patients, found an increase on WBC count of 42.7% in the ERAS group and an increase of 72.9% in the control group. Time required for the WBC count to normalize was significantly shorter in the ERAS group than in the control group (*p* ≤ 0.001). Tian et al. (19), 2020, including 1,026 patients. Although results were not quantitatively available in absolute numbers, a graphic representation suggests that WBC did not increase as much in the ERAS group as it did in the control. Both randomized control trials by Peng et al. (22), including 130 patients, and Mari et al. (20), including 140 patients, found no significant reduction of WBC in the ERAS group compared to the control group.

*Interleukin-6 (IL-6):* Two RCTs (18, 20) evaluated the impact of the ERAS protocol on patients undergoing laparoscopic surgery and the IL-6 marker. A total population of 303 patients, both studies found significantly lower levels of IL-6 on POD 3 after ERAS protocol groups vs. standard groups.

## Discussion

This scoping review evaluates the impact of Enhanced Recovery After Surgery (ERAS) recommendation measures during the perioperative period on inflammatory markers in patients undergoing laparoscopic surgery. It aims to elucidate consistent findings, recognize knowledge gaps, and suggest directions for future research on both existing and novel inflammatory markers.

The initial studies following the conceptualization of ERAS by Kehlet et al. (6) focused predominantly on morbidity, mortality, and surgical complications, rather than on the quantitative assessment of inflammatory markers. It took 13 years from the inception of this concept for the first study analyzing the influence of ERAS on inflammatory markers in laparoscopic surgeries to be published.

According to literature, open surgery, amongst several other factors, such as age >70 years, BMI ≥30 kg/m^2^, ASA score >2, dirty/contaminated surgery, as well as comorbidities (diabetes and chronic steroid use) were associated with significantly higher incidence of surgical site infection (SSI) (23, 24).

Results from literature regarding index admission and total expenses, including 30-day readmissions, demonstrated that laparoscopic approach is less expensive than open surgery and with shorter hospital length of stay (25).

Laparoscopic colorectal surgery was the most examined procedure, covered in five of the seven included studies. These studies collectively analyzed a total of 2,047 patients, with 891 undergoing colorectal procedures. The largest study, by Tian et al. (19), involved 1,026 patients and examined laparoscopic gastrectomy.

White blood cell count, and C-reactive protein were the most frequently assessed markers, each studied in four investigations. Notably, critical inflammatory markers such as tumor necrosis factor-alpha and alpha 1-acid glycoprotein were absent from the studies reviewed, representing a significant gap in the literature. Markers such as neutrophil gelatinase-associated lipocalin (NGAL) and N-terminal pro B-type natriuretic peptide (NT-proBNP), which indicate organ damage, were also not evaluated.

An innovative strategy that can be incorporated into perioperative optimization protocols is the adoption of probiotics in the perioperative period, a topic that was studied by Xiong et al. (26) In the 2023 (27) and 2024 study (26), patients undergoing radical distal gastrectomy were divided into two groups, probiotics vs. placebo. The group administered probiotics reduced the post-operative inflammatory response, accelerated the fall in the neutrophil-lymphocyte ratio (NLR), and promoted a faster increase in serum albumin concentration, further studies should be conducted to possibly add another tool to ERAS.

Inflammatory markers can also be useful for predicting postoperative complications, which can delay early discharge, one of the pillars of the perioperative optimization scenario. The study by Van Daele et al. (28) analyzed the diagnostic accuracy of biomarkers for anastomotic fistula in patients undergoing esophagectomy. When analyzing 5,348 patients (28), C-reactive protein was found to be a reliable negative predictor, indicating a low probability of fistula and supporting safe early discharge in the ERAS protocol.

All reviewed studies reported benefits of the ERAS recommendations over standard care, notably in reducing the surgical stress response, as evidenced by lower levels of IL-6, CRP, and WBCs. Moreover, a trend towards fewer postoperative complications was observed in ERAS patients, although statistical significance was achieved in only one study.

Our findings support the literature showing a beneficial impact of ERAS recommendations, particularly noted in significant reductions of CRP levels on postoperative days three and five (Figures 2, 3). However, the overall certainty of this evidence remains very low due to issues with precision, consistency, and directness.

This review's strengths lie in its methodical approach, including a comprehensive search, systematic selection, and rigorous bias assessment, independently replicated by multiple reviewers. Additionally, the GRADE approach was employed to enhance the reliability of evidence evaluation. Conversely, the primary limitations stem from high variability in study outcomes, insufficient blinding of surgical teams, and the reliance on a limited number of small-scale studies, which collectively restrict the precision and applicability of the findings.

This scoping review highlights the need for additional randomized controlled trials (RCTs) to more precisely determine the effects and validate the impact of Enhanced Recovery After Surgery (ERAS) recommendations on inflammatory markers in laparoscopic surgery. The preliminary findings suggest that the implementation of ERAS guidelines may significantly reduce inflammatory markers, potentially leading to enhanced recovery outcomes for patients undergoing laparoscopic procedures. However, due to the limited number of studies and their small sample sizes, the certainty of these findings remains very low, emphasizing the importance of further high-quality research to provide more definitive evidence and refine clinical practice guidelines.
